# 2-De­oxy-α-d-*arabino*-hexopyran­ose

**DOI:** 10.1107/S1600536811035264

**Published:** 2011-09-14

**Authors:** David Hess, Peter Klüfers

**Affiliations:** a Ludwig-Maximilians-Universität, Department Chemie und Biochemie, Butenandtstrasse 5–13, 81377 München, Germany

## Abstract

The title compound, C_6_H_12_O_5_, is the α-pyran­ose form of the reducing aldose 2-de­oxy-d-*arabino*-hexose. The six-membered pyran­ose ring adopts a ^4^ *C*_1_ conformation, with the anomeric hy­droxy group in axial and the other substituents in equatorial positions. In the crystal, each of the four hy­droxy groups acts as an inter­molecular hydrogen-bond donor function, resulting in a three-dimensional hydrogen-bonded network.

## Related literature

For the crystal structure of 2-de­oxy-β-d-*arabino*-hexopyran­ose, see: Maluszynska *et al.* (1981 ▶) and for the crystal structures of α-d-glucose and α-d-mannose, see Brown *et al.* (1965 ▶) and Longchambon *et al.* (1976 ▶), respectively. For puckering parameters, see: Cremer & Pople (1975 ▶). Crystals of the title compound were obtained during the course of attemps to grow crystals of a phenyl­boronic acid ester of 2-de­oxy-d-*arabino*-hexose, see: Hess & Klüfers (2011 ▶).
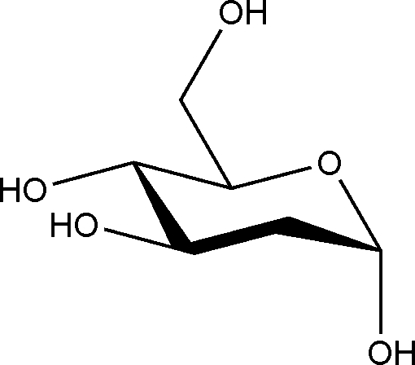


## Experimental

### 

#### Crystal data

C_6_H_12_O_5_
*M* *_r_* = 164.16
Orthorhombic, 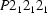

*a* = 4.8538 (2) Å
*b* = 9.5323 (4) Å
*c* = 15.6718 (6) Å
*V* = 725.12 (5) Å^3^
*Z* = 4
Mo *K*α radiation
μ = 0.13 mm^−1^
*T* = 200 K
0.21 × 0.06 × 0.05 mm

#### Data collection

Nonius KappaCCD diffractometer
5622 measured reflections
1001 independent reflections
937 reflections with *I* > 2σ(*I*)
*R*_int_ = 0.030

#### Refinement

*R*[*F* ^2^ > 2σ(*F* ^2^)] = 0.035
*wR*(*F* ^2^) = 0.097
*S* = 1.14
1001 reflections
104 parameters
H-atom parameters constrained
Δρ_max_ = 0.43 e Å^−3^
Δρ_min_ = −0.19 e Å^−3^

### 

Data collection: *COLLECT* (Nonius, 2004 ▶); cell refinement: *SCALEPACK* (Otwinowski & Minor, 1997 ▶); data reduction: *DENZO* (Otwinowski & Minor, 1997 ▶) and *SCALEPACK*; program(s) used to solve structure: *SHELXS97* (Sheldrick, 2008 ▶); program(s) used to refine structure: *SHELXL97* (Sheldrick, 2008 ▶); molecular graphics: *ORTEP-3* (Farrugia, 1997 ▶) and *SCHAKAL99* (Keller, 1999 ▶); software used to prepare material for publication: *SHELXL97*.

## Supplementary Material

Crystal structure: contains datablock(s) I, global. DOI: 10.1107/S1600536811035264/kj2184sup1.cif

Structure factors: contains datablock(s) I. DOI: 10.1107/S1600536811035264/kj2184Isup2.hkl

Additional supplementary materials:  crystallographic information; 3D view; checkCIF report

## Figures and Tables

**Table 1:** Hydrogen-bond geometry (Å, °)

*D*—H⋯*A*	*D*—H	H⋯*A*	*D*⋯*A*	*D*—H⋯*A*
O1—H81⋯O5^i^	0.84	1.95	2.780 (2)	171
O3—H83⋯O1^ii^	0.84	2.00	2.784 (2)	155
O4—H84⋯O6^iii^	0.84	1.94	2.776 (3)	174
O6—H86⋯O3^iv^	0.84	1.84	2.670 (2)	170

Symmetry codes: (i) 
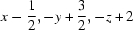
; (ii) 
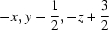
; (iii) 
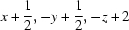
; (iv) 
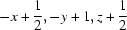
.

**Fig. 1.**
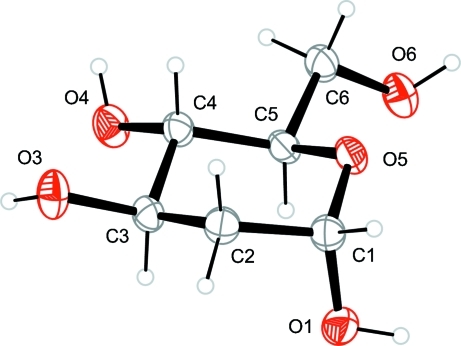
ORTEP-representation of the asymmetric unit of the title compound, with atom labels and anisotropic displacement ellipsoids (drawn at 50% probability level) for non-H atoms.

**Fig. 2.**
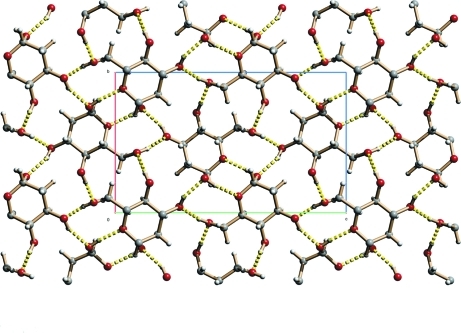
SCHAKAL-representation of hydrogen bonds in the crystal packing of the title compound viewed along the a axis.

## supplementary crystallographic information

### Comment

2-Deoxy-D-*arabino*-hexose is the 2-deoxy derivate of both D-glucose and D-mannose. The crystals of the title compound were obtained in the course of attemps to grow crystals of a phenylboronic acid ester of 2-deoxy-D-*arabino*-hexose (Hess & Klüfers, 2011).

The bond lenghts and angles between the non-hydrogen atoms are normal. The pyranose ring adopts a slightly distorted ^4^C_1_ conformation, the puckering parameters (Cremer & Pople, 1975) being *Q* = 0.551 (2) Å and θ = 6.0 (2)° (Fig. 1). The exocyclic C6—O6 bond is orientated *gauche*-*trans* relative to the C5—O5 and C4—C5 bonds of the ring. In the crystal structure the compound forms a three-dimensional hydrogen-bonded network, where each hydroxy group acts as a donor in an intermolecular hydrogen bond to a different neighboring molecule. Acceptor functions are either the ring oxygen atom (O5) or the hydroxy oxygen atoms (O1, O3, O6). The hydrogen bond pattern is shown in Figure 2.

### Experimental

2-Deoxy-D-*arabino*-hexose (0.164 g, 1 mmol) was dissolved in 1 ml of water and a solution of phenylboronic acid (0.122 g, 1 mmol) in 1 ml of methanol was added. The obtained solution was stirred at ambient temperature for 2 h. The solvent was then removed under reduced pressure. The remaining solid was dissolved in aceton and slowly evaporated to give colourless crystals suitable for X-ray analysis.

### Refinement

Since the compound is a weak anomalous scatterer, 662 Friedel pairs were merged. The absolute structure was assigned according to the known stereochemistry of the starting material. Carbon-bound as well as oxygen-bound H atoms were placed in calculated positions (C—H 0.99 Å for CH_2_-groups, C—H 1.00 Å for CH-groups and O—H 0.84 Å for hydoxy groups) and were included in the refinement in the riding model approximation, with *U*_iso_(H) set to 1.2*U*_eq_(C) for the CH2-groups and CH-groups and 1.5*U*_eq_(O) for the hydroxy groups.

### Crystal data

**Table:**

C_6_H_12_O_5_	*F*(000) = 352
*M**_r_* = 164.16	*D*_x_ = 1.504 (1) Mg m^−^^3^
Orthorhombic, *P*2_1_2_1_2_1_	Mo *K*α radiation, λ = 0.71073 Å
Hall symbol: P 2ac 2ab	Cell parameters from 2672 reflections
*a* = 4.8538 (2) Å	θ = 3.1–27.5°
*b* = 9.5323 (4) Å	µ = 0.13 mm^−^^1^
*c* = 15.6718 (6) Å	*T* = 200 K
*V* = 725.12 (5) Å^3^	Rod, colourless
*Z* = 4	0.21 × 0.06 × 0.05 mm

### Data collection

**Table:**

Nonius KappaCCD diffractometer	937 reflections with *I* > 2σ(*I*)
Radiation source: rotating anode	*R*_int_ = 0.030
MONTEL, graded multilayered X-ray optics	θ_max_ = 27.5°, θ_min_ = 3.4°
CCD; rotation images; thick slices scans	*h* = −6→6
5622 measured reflections	*k* = −12→12
1001 independent reflections	*l* = −20→20

### Refinement

**Table:**

Refinement on *F*^2^	Primary atom site location: structure-invariant direct methods
Least-squares matrix: full	Secondary atom site location: difference Fourier map
*R*[*F*^2^ > 2σ(*F*^2^)] = 0.035	Hydrogen site location: inferred from neighbouring sites
*wR*(*F*^2^) = 0.097	H-atom parameters constrained
*S* = 1.14	*w* = 1/[σ^2^(*F*_o_^2^) + (0.0454*P*)^2^ + 0.298*P*] where *P* = (*F*_o_^2^ + 2*F*_c_^2^)/3
1001 reflections	(Δ/σ)_max_ < 0.001
104 parameters	Δρ_max_ = 0.43 e Å^−^^3^
0 restraints	Δρ_min_ = −0.19 e Å^−^^3^

### Fractional atomic coordinates and isotropic or equivalent isotropic displacement parameters (Å^2^)

**Table:**

	*x*	*y*	*z*	*U*_iso_*/*U*_eq_	
O1	−0.0906 (4)	0.75609 (18)	0.88687 (10)	0.0296 (4)	
H81	−0.1430	0.8030	0.9292	0.044*	
O3	0.3063 (4)	0.45829 (17)	0.72256 (9)	0.0308 (4)	
H83	0.2179	0.3892	0.7036	0.046*	
O4	0.0700 (4)	0.28748 (16)	0.85187 (10)	0.0292 (4)	
H84	0.1642	0.2202	0.8705	0.044*	
O5	0.2069 (4)	0.61635 (15)	0.96649 (9)	0.0251 (4)	
O6	−0.0846 (4)	0.42652 (18)	1.09339 (9)	0.0305 (4)	
H86	−0.0049	0.4550	1.1377	0.046*	
C1	0.1847 (6)	0.7158 (2)	0.89833 (14)	0.0259 (5)	
H1	0.2957	0.8008	0.9132	0.031*	
C2	0.2911 (5)	0.6559 (2)	0.81511 (13)	0.0248 (5)	
H2A	0.2463	0.7213	0.7681	0.030*	
H2B	0.4941	0.6473	0.8182	0.030*	
C3	0.1683 (5)	0.5136 (2)	0.79562 (12)	0.0229 (5)	
H3	−0.0325	0.5243	0.7828	0.027*	
C4	0.2049 (5)	0.4156 (2)	0.87086 (12)	0.0219 (4)	
H4	0.4055	0.3980	0.8806	0.026*	
C5	0.0780 (5)	0.4826 (2)	0.95053 (12)	0.0219 (4)	
H5	−0.1235	0.4975	0.9406	0.026*	
C6	0.1161 (5)	0.3944 (2)	1.02968 (13)	0.0263 (5)	
H6A	0.1017	0.2939	1.0143	0.032*	
H6B	0.3026	0.4108	1.0532	0.032*	

### Atomic displacement parameters (Å^2^)

**Table:**

	*U*^11^	*U*^22^	*U*^33^	*U*^12^	*U*^13^	*U*^23^
O1	0.0353 (9)	0.0299 (8)	0.0238 (7)	0.0080 (8)	0.0022 (7)	0.0000 (7)
O3	0.0436 (10)	0.0274 (8)	0.0214 (7)	−0.0055 (8)	0.0082 (8)	−0.0041 (6)
O4	0.0396 (9)	0.0213 (7)	0.0269 (8)	−0.0068 (8)	−0.0051 (8)	−0.0013 (6)
O5	0.0372 (9)	0.0192 (7)	0.0189 (6)	−0.0015 (7)	−0.0060 (7)	−0.0002 (6)
O6	0.0379 (9)	0.0340 (9)	0.0195 (7)	−0.0042 (8)	0.0005 (7)	−0.0010 (6)
C1	0.0346 (12)	0.0215 (10)	0.0216 (9)	0.0010 (10)	−0.0013 (10)	0.0013 (8)
C2	0.0297 (11)	0.0221 (10)	0.0226 (9)	−0.0003 (10)	0.0013 (10)	0.0023 (8)
C3	0.0261 (11)	0.0256 (10)	0.0170 (8)	0.0004 (10)	0.0017 (8)	−0.0003 (8)
C4	0.0261 (10)	0.0186 (10)	0.0211 (9)	−0.0016 (9)	−0.0034 (9)	−0.0015 (7)
C5	0.0265 (10)	0.0206 (9)	0.0186 (9)	−0.0012 (9)	−0.0049 (9)	0.0003 (7)
C6	0.0357 (12)	0.0239 (10)	0.0192 (9)	0.0020 (10)	−0.0017 (9)	0.0010 (8)

### Geometric parameters (Å, °)

**Table:**

O1—C1	1.402 (3)	C2—C3	1.512 (3)
O1—H81	0.8400	C2—H2A	0.9900
O3—C3	1.428 (2)	C2—H2B	0.9900
O3—H83	0.8400	C3—C4	1.515 (3)
O4—C4	1.418 (3)	C3—H3	1.0000
O4—H84	0.8400	C4—C5	1.532 (3)
O5—C1	1.433 (3)	C4—H4	1.0000
O5—C5	1.442 (3)	C5—C6	1.510 (3)
O6—C6	1.428 (3)	C5—H5	1.0000
O6—H86	0.8400	C6—H6A	0.9900
C1—C2	1.515 (3)	C6—H6B	0.9900
C1—H1	1.0000		
			
?···?	?		
			
C1—O1—H81	109.5	C2—C3—H3	109.5
C3—O3—H83	109.5	C4—C3—H3	109.5
C4—O4—H84	109.5	O4—C4—C3	108.28 (16)
C1—O5—C5	115.04 (15)	O4—C4—C5	110.16 (18)
C6—O6—H86	109.5	C3—C4—C5	109.27 (17)
O1—C1—O5	110.39 (19)	O4—C4—H4	109.7
O1—C1—C2	108.54 (19)	C3—C4—H4	109.7
O5—C1—C2	111.50 (18)	C5—C4—H4	109.7
O1—C1—H1	108.8	O5—C5—C6	107.26 (16)
O5—C1—H1	108.8	O5—C5—C4	109.60 (18)
C2—C1—H1	108.8	C6—C5—C4	112.82 (17)
C3—C2—C1	112.20 (18)	O5—C5—H5	109.0
C3—C2—H2A	109.2	C6—C5—H5	109.0
C1—C2—H2A	109.2	C4—C5—H5	109.0
C3—C2—H2B	109.2	O6—C6—C5	111.79 (18)
C1—C2—H2B	109.2	O6—C6—H6A	109.3
H2A—C2—H2B	107.9	C5—C6—H6A	109.3
O3—C3—C2	107.95 (17)	O6—C6—H6B	109.3
O3—C3—C4	109.96 (17)	C5—C6—H6B	109.3
C2—C3—C4	110.45 (16)	H6A—C6—H6B	107.9
O3—C3—H3	109.5		
			
C5—O5—C1—O1	66.3 (2)	C2—C3—C4—C5	56.0 (2)
C5—O5—C1—C2	−54.4 (3)	C1—O5—C5—C6	−178.49 (18)
O1—C1—C2—C3	−71.6 (2)	C1—O5—C5—C4	58.7 (2)
O5—C1—C2—C3	50.2 (3)	O4—C4—C5—O5	−176.95 (16)
C1—C2—C3—O3	−172.65 (19)	C3—C4—C5—O5	−58.1 (2)
C1—C2—C3—C4	−52.4 (2)	O4—C4—C5—C6	63.6 (2)
O3—C3—C4—O4	−65.0 (2)	C3—C4—C5—C6	−177.56 (19)
C2—C3—C4—O4	175.95 (18)	O5—C5—C6—O6	81.9 (2)
O3—C3—C4—C5	174.99 (18)	C4—C5—C6—O6	−157.32 (19)

### Hydrogen-bond geometry (Å, °)

**Table:**

*D*—H···*A*	*D*—H	H···*A*	*D*···*A*	*D*—H···*A*
O1—H81···O5^i^	0.84	1.95	2.780 (2)	171.
O3—H83···O1^ii^	0.84	2.00	2.784 (2)	155.
O4—H84···O6^iii^	0.84	1.94	2.776 (3)	174.
O6—H86···O3^iv^	0.84	1.84	2.670 (2)	170.

Symmetry codes: (i) *x*−1/2, −*y*+3/2, −*z*+2; (ii) −*x*, *y*−1/2, −*z*+3/2; (iii) *x*+1/2, −*y*+1/2, −*z*+2; (iv) −*x*+1/2, −*y*+1, *z*+1/2.
